# Comparison of the modified Singapore myocardial infarction registry risk score with GRACE 2.0 in predicting 1-year acute myocardial infarction outcomes

**DOI:** 10.1038/s41598-022-16523-6

**Published:** 2022-08-22

**Authors:** Ching-Hui Sia, Huili Zheng, Junsuk Ko, Andrew Fu-Wah Ho, David Foo, Ling-Li Foo, Patrick Zhan-Yun Lim, Boon Wah Liew, Ping Chai, Tiong-Cheng Yeo, Huay-Cheem Tan, Terrance Chua, Mark Yan-Yee Chan, Jack Wei Chieh Tan, Keith A. A. Fox, Heerajnarain Bulluck, Derek J. Hausenloy

**Affiliations:** 1 Department of Cardiology, National University Heart Centre Singapore, Singapore, Singapore; 2 Yong Loo Lin School of Medicine, National University of Singapore, Singapore, Singapore; 3 Health Promotion Board, National Registry of Diseases Office, Singapore, Singapore; 4 MD Program, Duke-NUS Medical School, Singapore, Singapore; 5 SingHealth Duke-NUS Emergency Medicine Academic Clinical Programme, Singapore, Singapore; 6 National Heart Research Institute Singapore, National Heart Centre Singapore, Singapore, Singapore; 7 Pre-Hospital and Emergency Care Research Centre, Health Services and Systems Research, Duke-NUS Medical School, Singapore, Singapore; 8 Tan Tock Seng Hospital, Singapore, Singapore; 9 Khoo Teck Puat Hospital, Singapore, Singapore; 10 Changi General Hospital, Singapore, Singapore; 11 Department of Cardiology, National Heart Centre Singapore, Singapore, Singapore; 12 Centre for Cardiovascular Science, University of Edinburgh, Edinburgh, UK; 13 Leeds Teaching Hospital NHS trust, Leeds, UK; 14 Cardiovascular and Metabolic Disorders Program, Duke-National University of Singapore Medical School, 8 College Road, Level 8, Singapore, 169857 Singapore; 15 The Hatter Cardiovascular Institute, University College London, London, UK; 16 Cardiovascular Research Center, College of Medical and Health Sciences, Asia University, Taichung City, Taiwan

**Keywords:** Cardiology, Medical research, Risk factors

## Abstract

Risk stratification plays a key role in identifying acute myocardial infarction (AMI) patients at higher risk of mortality. However, current AMI risk scores such as the Global Registry of Acute Coronary Events (GRACE) score were derived from predominantly Caucasian populations and may not be applicable to Asian populations. We previously developed an AMI risk score from the national-level Singapore Myocardial Infarction Registry (SMIR) confined to ST-segment elevation myocardial infarction (STEMI) patients and did not include non-STEMI (NSTEMI) patients. Here, we derived a modified SMIR risk score for both STEMI and NSTEMI patients and compared its performance to the GRACE 2.0 score for predicting 1-year all-cause mortality in our multi-ethnic population. The most significant predictor of 1-year all-cause mortality in our population using the GRACE 2.0 score was cardiopulmonary resuscitation on admission (adjusted hazards ratio [HR] 6.50), while the most significant predictor using the SMIR score was age 80–89 years (adjusted HR 7.78). Although the variables used in the GRACE 2.0 score and SMIR score were not exactly the same, the c-statistics for 1-year all-cause mortality were similar between the two scores (GRACE 2.0 0.841 and SMIR 0.865). In conclusion, we have shown that in a multi-ethnic Asian AMI population undergoing PCI, the SMIR score performed as well as the GRACE 2.0 score.

## Introduction

Mortality and morbidity remain significant in patients with acute myocardial infarction (AMI)^1^. As patients vary in prognosis, it is crucial to determine which patients are expected to perform poorly so that aggressive treatment can be targeted towards that group of patients^2,3^. Risk stratification plays a key role in identifying the high-risk patients. Several risk scores have been developed, including the Global Registry of Acute Coronary Events (GRACE) and Thrombolysis In Myocardial infarction (TIMI) scores, but these scores were primarily derived from and validated in mainly Caucasian populations^2–4^. A previous study performed in Singapore showed that the GRACE score underestimated in-hospital mortality after AMI in a multi-ethnic Asian population^1^.

The most recent American College of Cardiology/American Heart Association and European Society of Cardiology guidelines recommended the use of GRACE and TIMI scores for risk stratification^5,6^, and there are emerging studies evaluating whether these scores apply to ethnically homogenous Asian populations^7,8^. The Korean Acute Myocardial Infarction Registry (KAMIR) score was created to risk stratify the Korean AMI population^9^. However, the performance of the scores developed for the Caucasian and Korean populations may not be applicable to the Singaporean population as the latter population is multi-ethnic^2^. Furthermore, it is uncertain how the previously developed SMIR risk score for ST-segment elevation myocaridal infarction (STEMI) patients compares to the guideline-recommended GRACE score in predicting 1-year all-cause mortality^2^. The SMIR risk score was previously used for predicting in-hospital, 30-day and 1-year cardiac mortality, as well as 1-year heart failure rehospitalization, but not for 1-year all-cause mortality. The previous SMIR risk score was also not meant to risk stratify non-ST segment elevation myocardial infarction (NSTEMI) patients.

As such, we sought to develop a new SMIR score and evaluate the performance of both the modified SMIR and GRACE 2.0 scores in predicting 1-year all-cause mortality among a population-based real-world multi-ethnic Asian STEMI and NSTEMI population with percutaneous coronary intervention (PCI).

## Methods

### Data collection

We utilized data from the Singapore Myocardial Infarction Registry (SMIR) for this study. SMIR is a national, ministry-funded registry run by the National Registry of Diseases Office (NRDO). The local ethics committee granted an exemption review for this study (SingHealth CIRB Reference No: 2016/2480) with a waiver of need for informed consent as the study utilised de-identified data. The study was performed in accordance with the Declaration of Helsinki. The statistician could access the anonymised individual-level data, while the rest of the co-authors could only access the analysed aggregated data.

SMIR obtains clinical data of all AMI patients from the public and private hosptials in Singapore^10–14^. Healthcare practitioners are mandated by law to notify the registry of the AMI cases, based on the International Classification of Diseases, 10th Revision (Australian Modification) codes I21 and I22. Patients’ were notified through medical claim listings, patient discharge summaries and laboratory results, while patients’ data were extracted from their medical records by the registry co-ordinators. These clinical data were then merged with the death data from the Registry of Births and Deaths. The Registry of Births and Deaths captures all mortality outcomes in Singapore through mandatory reporting. The registry data was subject to annual audits for accuracy and inter-rater reliability. Outlier and illogical data were flagged for review. We looked at STEMI patients who underwent primary percutaneous coronary intervention (PPCI) and NSTEMI patients who underwent PCI. AMI patients treated medically without PCI were excluded as their clinical characteristics were heterogenous.

### Derivation of GRACE 2.0 score and SMIR score

The GRACE 2.0 score, derived from the GRACE registry involving 94 hospitals from 14 countries, was an improved and preferred version refined from the original GRACE score. This score was validated in the French registry of STEMI and NSTEMI (FAST-MI). A higher GRACE 2.0 score is associated with a higher mortality risk up to 3 years after the initial acute coronary syndrome event^3^. We chose to study the GRACE 2.0 score instead of the original GRACE score as this improved version could predict mortality beyond the initial hospitalization^3^.

Using SMIR data of STEMI patients with PPCI and NSTEMI patients with PCI in January 2017 to June 2018 and the same method previously used to derive the original SMIR score for STEMI patients^2^, we developed the modified SMIR score whereby a random sample of 70% of the cases (n = 3960) were used to derive the score and the remaining 30% (n = 1698) were used to validate the score. The components in the modified SMIR score were: age at onset of AMI, history of diabetes, Killip class on admission, cardiopulmonary resuscitation (CPR) on admission, systolic blood pressure on admission, creatinine on admission, haemoglobin on admission and left ventricular ejection fraction (LVEF) during hospitalization. Supplementary Table 1 shows the score allocation of the components and the predicted risk from the modified SMIR score. While the derivation of the modified SMIR score was largely driven by the SMIR data, it also used empirical evidence to categorize the numeric variables in the score. While using continuous functions to handle the numeric variables like the GRACE 2.0 score might yield better predictive ability, we handled the numeric variables in the modified SMIR score using a categorical approach so that the interpretation of the predicted risk contributed by each variable in the score would be easier. Unlike the previous study by Chan et al.^1^, we did not re-calibrate the GRACE 2.0 score to fit the local AMI population as we were keen to consider other variables that might be crucial in risk prediction.

### Statistical analysis

As the modified SMIR score was validated on a randomly selected 30% of the STEMI patients with PPCI and NSTEMI patients with PCI in January 2017 to June 2018 from SMIR (n = 1698), we calculated the GRACE 2.0 score on the same group of patients and compared the performance of the two scores. We did not look at patients with PCI prior to January 2017 as SMIR only started to capture heart rate and blood pressure, variables included in the GRACE 2.0 score, from 2017 onwards. We also did not apply the scores on patients with PCI after June 2018 as the death data available at the point of analysis was until June 2019 and our outcome of interest was 1-year all-cause mortality.

The demographics and clinical chracteristics of all the AMI patients included in this study were expressed as frequency with percentages for categorical variables and median with interquartile range continuous variables. Cox regression was performed to determine the hazards ratios of the components of the GRACE 2.0 and modified SMIR scores. Patients were divided into groups based on their predicted risk of 1-year all-cause mortality from the GRACE 2.0 and modified SMIR scores. Actual mortality among the patients in each group was calculated to see if the observed mortality were close to the predicted mortality estimated from the two scores. The receiver operator chracteristic (ROC) curve of each score was plotted and the area under the curves were compared to see how well each score predicted 1-year all-cause mortality. The same analyses were replicated for each of the three main ethnic groups in Singapore to see if the performance of the two scores differed by ethnic group. Missing data were excluded from the analyses through case deletion without imputation to maintain data in its original form. All statistical analyses were performed using Stata (StataCorp. 2013. *Stata Statistical Software: Release 13*. College Station, TX: StataCorp LP). All statistical tests were 2-tailed and results were deemed to be statistically significant if p < 0.05.

## Results

### Baseline characteristics

Our study included 5658 patients from January 2017 to June 2018. Baseline characteristics of the included patients are described in Table 1. The median age of the patients was 61.2 years (IQR 54.0, 69.6), and they were predominantly male (81.1%). Our study consisted of a multi-ethnic Asian population, whereby the majority of patients were Chinese (62.4%). The most common co-morbidity among the patients was hypertension (61.8%), followed by hyperlipidaemia (58.4%) and then diabetes (37.6%). There were more smokers (current 38.9%, former 17.7%) than non-smokers (43.3%). Most patients were Killip Class I on admission (83.8%). The majority of patients were on evidence-based therapies for AMI during hospitalization.

**Table 1:** Baseline characteristics of all acute myocardial infarction patients included in this study (n = 5658).

**Demographics**	
Age in years, median (IQR)	61.2 (54.0–69.6)
Male, n (%)	4587 (81.1)
Race, n (%)	
Chinese	3532 (62.4)
Malay	1063 (18.8)
Indian	969 (17.1)
Others	94 (1.7)
**Risk factors**	
History of hypertension, n (%)	3495 (61.8)
History of diabetes, n (%)	2126 (37.6)
History of hyperlipidemia, n (%)	3303 (58.4)
History of MI/PCI/CABG, n (%)	1431 (25.3)
Smoking status, n (%)	
Current	2192 (38.9)
Former	998 (17.7)
Never	2440 (43.3)
Killip class on admission, n (%)	
I	4737 (83.8)
II	299 (5.3)
III	353 (6.2)
IV	263 (4.7)
CPR on admission, n (%)	193 (3.4)
Heart rate in BPM on admission, median (IQR)	79 (67–93)
Systolic blood pressure in mmHg on admission, median (IQR)	135 (116–155)
Abnormal cardiac enzymes within 72 h from MI onset, n (%)	4743 (84.2)
Serum creatinine in µmol on admission, median (IQR)	90 (76–112)
Haemoglobin in g/dL on admission, median (IQR)	14.2 (12.7–15.3)
Treatment during hospitalization	
Aspirin, n (%)	5469 (96.7)
Beta blocker, n (%)	4886 (86.4)
ACEI/ARB, n (%)	3980 (70.3)
Lipid lowering drug, n (%)	5486 (97.0)
P2Y12 inhibitor, n (%)	5557 (98.2)

*ACEI/ARB* angiotensin-receptor converting enzyme inhibitor/angiotensin receptor blocker, *BPM* beats per minute, *CABG* coronary artery bypass grafting, *CPR* cardiopulmonary resuscitation, *ED* emergency department, *IQR* interquartile range, *MI* myocardial infarction, *PCI* percutaneous coronary intervention.

### Comparison of GRACE 2.0 and modified SMIR scores based on patients in SMIR

The unadjusted and adjusted hazard ratios (HR) of the individual components of the GRACE 2.0 and modified SMIR scores are shown in Table 2. For the GRACE 2.0 score, the three most significant predictors of 1-year all-cause mortality were CPR on admission (adjusted HR 6.50, 95% CI 3.82–11.06), high Killip Class on admission (adjusted HR for Class IV 4.98, 95% CI 3.14–7.91) and increasing age per 10 years (adjusted HR 1.70, 95% CI 1.45–1.99). Increasing systolic blood pressure per 20 mmHg on admission was protective (adjusted HR 0.84, 95% CI 0.75–0.95). For the modified SMIR score, the three most significant predictors of 1-year all-cause mortality were old age (adjusted HR for 70–79 years 3.53, 95% CI 1.27–9.81; adjusted HR for 80–89 years 7.78, 95% CI 2.68–22.57), CPR on admission (adjusted HR 6.34, 95% CI 3.35–12.00) and high Killip Class on admission (adjusted HR for Class IV 3.02, 95% CI 1.72–5.31). A higher LVEF during hospitalization was protective.

**Table 2:** Unadjusted and adjusted hazards ratios for the individual components of the GRACE 2.0 and modified SMIR scores among the AMI patients in SMIR (n = 1698).

	GRACE 2.0 score		Modified SMIR score	
	Unadjusted HR (95% CI)	Adjusted HR (95% CI)	Unadjusted HR (95% CI)	Adjusted HR (95% CI)
**Demographics**				
Age				
Per 10 years	1.63 (1.41–1.87)	1.70 (1.45–1.99)		
< 40 years			NA	NA
40–49 years			1.00 (ref)	1.00 (ref)
50–59 years			2.23 (0.93–5.32)	2.05 (0.76–5.52)
60–69 years			3.10 (1.32–7.28)	2.49 (0.93–6.67)
70–79 years			4.96 (2.08–11.78)	3.53 (1.27–9.81)
80–89 years			8.66 (3.54–21.19)	7.78 (2.68–22.57)
≥ 90 years			5.23 (0.63–43.43)	5.99 (0.66–54.32)
**Risk factors**				
History of diabetes			2.47 (1.77–3.45)	2.22 (1.45–3.40)
Killip class on admission				
I	1.00 (ref)	1.00 (ref)	1.00 (ref)	1.00 (ref)
II	2.86 (1.65–4.99)	1.86 (1.03–3.37)	2.86 (1.65–4.99)	1.04 (0.54–1.99)
III	3.64 (2.17–6.08)	2.64 (1.55–4.49)	3.64 (2.17–6.08)	0.95 (0.50–1.81)
IV	10.71 (7.14–16.08)	4.98 (3.14–7.91)	10.71 (7.14–16.08)	3.02 (1.72–5.31)
CPR on admission	8.55 (5.54–13.17)	6.50 (3.82–11.06)	8.55 (5.54–13.17)	6.34 (3.35–12.00)
Heart rate on admission				
Per 30 BPM	1.13 (1.07–1.18)	1.11 (1.04–1.18)		
< 50 BPM				
50–69 BPM				
70–79 BPM				
80–89 BPM				
90–99 BPM				
100–109 BPM				
110–129 BPM				
130–149 BPM				
≥ 150 BPM				
Systolic blood pressure on admission				
Per 20 mmHg	0.75 (0.66–0.85)	0.84 (0.75–0.95)		
< 80 mmHg			1.00 (ref)	1.00 (ref)
80–99 mmHg			0.43 (0.21–0.87)	1.81 (0.66–4.94)
100–109 mmHg			0.29 (0.13–0.62)	1.93 (0.67–5.56)
110–119 mmHg			0.19 (0.09–0.41)	0.72 (0.25–2.08)
120–129 mmHg			0.11 (0.05–0.25)	0.73 (0.24–2.22)
130–139 mmHg			0.12 (0.06–0.28)	1.26 (0.43–3.67)
140–159 mmHg			0.13 (0.06–0.27)	0.79 (0.29–2.15)
160–179 mmHg			0.14 (0.06–0.31)	0.93 (0.30–2.93)
≥ 180 mmHg			0.18 (0.08–0.41)	0.85 (0.28–2.60)
Abnormal cardiac enzymes within 72 h from MI onset	1.10 (0.69–1.75)	0.74 (0.46–1.18)		
Serum creatinine on admission				
Per mg/dL	1.16 (1.11–1.20)	1.19 (1.13–1.24)		
< 35 µmol/L			1.00 (ref)	1.00 (ref)
35–69 µmol/L			0.07 (0.01–0.56)	0.06 (0.01–0.53)
70–105 µmol/L			0.07 (0.01–0.49)	0.06 (0.01–0.48)
106–140 µmol/L			0.24 (0.03–1.75)	0.11 (0.01–0.91)
141–176 µmol/L			0.48 (0.06–3.61)	0.16 (0.02–1.43)
177–353 µmol/L			0.63 (0.08–4.70)	0.22 (0.03–1.96)
≥ 354 µmol/L			0.49 (0.07–3.64)	0.16 (0.02–1.43)
Haemoglobin on admission				
< 10 g/dL			1.00 (ref)	1.00 (ref)
10–11 g/dL			0.65 (0.37–1.13)	0.69 (0.36–1.32)
12–13 g/dL			0.30 (0.17–0.51)	0.73 (0.38–1.42)
14–15 g/dL			0.16 (0.09–0.28)	0.41 (0.19–0.89)
≥ 16 g/dL			0.22 (0.11–0.42)	0.71 (0.31–1.67)
STEMI				
ST deviation	1.34 (0.94–1.91)	1.28 (0.86–1.91)		
LVEF during hospitalization				
< 30%			1.00 (ref)	1.00 (ref)
30–39%			0.41 (0.26–0.65)	0.56 (0.34–0.92)
40–49%			0.19 (0.11–0.32)	0.32 (0.18–0.56)
≥ 50%			0.10 (0.06–0.17)	0.23 (0.13–0.40)

*CI* confidence interval, *CPR* cardiopulmonary resuscitation, *ED* emergency department, *GRACE* Global Registry of Acute Coronary Events, *MI* myocardial infarction, *SMIR* Singapore Myocardial Infarction Registry.

**Figure 1:**
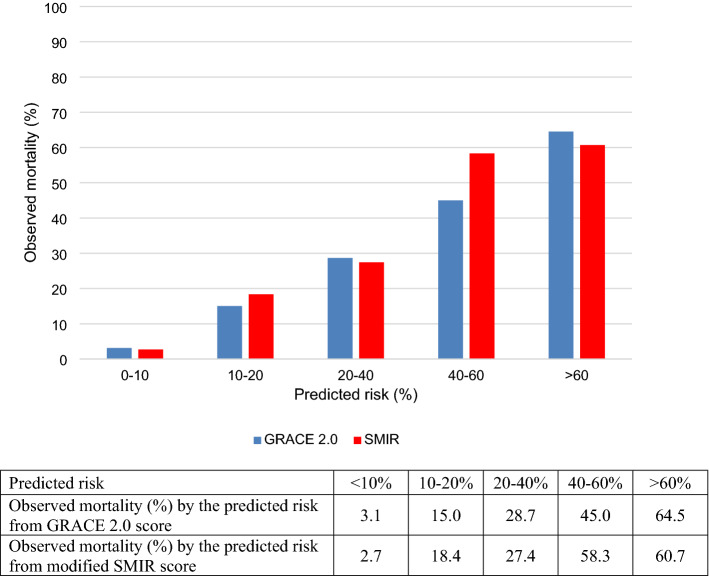
Observed 1-year all-cause mortality and predicted risk from the GRACE 2.0 and modified SMIR scores. The predicted mortality from the GRACE 2.0 (blue) and modified SMIR (red) scores were compared with the actual 1-year all-cause mortality observed among the AMI patients in SMIR. *GRACE* Global Registry of Acute Coronary Events, *SMIR* Singapore Myocardial Infarction Registry.

**Figure 2:**
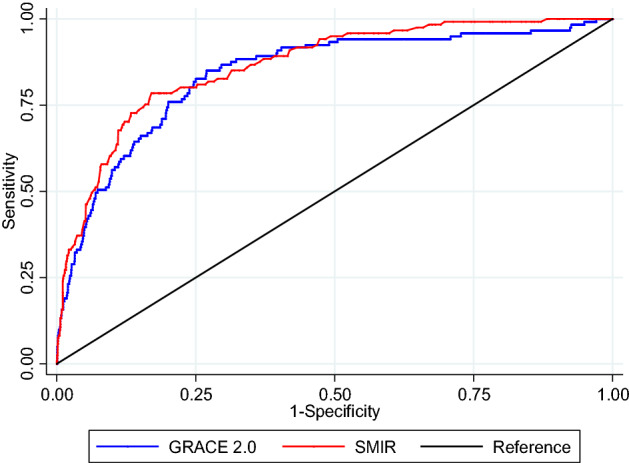
Receiver operator characteristics curves of the GRACE 2.0 and modified SMIR scores. The areas under the curve of the GRACE 2.0 (blue) and modified SMIR (red) scores were plotted and estimated to compare the performance of the two scores among the AMI patients in SMIR. AUC (95% CI) of GRACE 2.0 score: 0.841 (0.802–0.880). AUC (95% CI) of modified SMIR score: 0.865 (0.833–0.898). No statistically significant difference in AUC between the two scores: p = 0.075. *AUC* area under the curve, *CI* confidence interval, *GRACE* Global Registry of Acute Coronary Events, *SMIR* Singapore Myocardial Infarction Registry.

The predicted risk of 1-year all-cause death was < 10% for most of the patients based on the GRACE 2.0 and modified SMIR scores (Supplementary Table 2). To reduce statistical variability, we collapsed the patients into broader groups based on their predicted risk and looked at the actual observed mortality in each group. The observed mortality generally increased with the predicted mortality from both the GRACE 2.0 and modified SMIR scores, indicating positive correlation between the scores and actual outcome (Fig. 1). There was no statistically significant difference between the area under the curves for the two scores in predicting 1-year all-cause mortality (p = 0.075) (Fig. 2). The area under the ROC curve for the GRACE 2.0 score was 0.841 (95% CI 0.802–0.880) and that for the modified SMIR score was 0.865 (95% CI 0.833–0.898). Stratifying by the three main ethnic groups in Singapore, the performance of the two scores remained similar without statistically significant difference in area under the curves for predicting 1-year all-cause mortality (Supplementary Tables 3 and 4).

## Discussion

In this real-world population-based study, we showed that the modified SMIR score performed similarly to the GRACE 2.0 score in a multi-ethnic Asian population in predicting 1-year all-cause mortality following STEMI and NSTEMI.

Inter-ethnic differences in the outcomes of STEMI patients have been published previously. Previous studies performed both locally^15–17^ and abroad^18^ have suggested inter-ethnic differences in terms of outcomes such as mortality. While there are established coronary risk factors, such as smoking, hypertension, hyperlipidaemia and diabetes mellitus, these risk factors cannot fully account for the observed inter-ethnic variations in outcomes^19^. Ethnic differences also existed in possible pathophysiological factors such as economic, lifestyle, anthropometric, and patient susceptibility to cerebrovascular diseases^16,18^. Of note, these factors are not included in contemporary risk scores such as the TIMI and GRACE 2.0 scores^3,4^, and are also difficult to ascertain in the acute setting. As such, there is a need to assess the relevance of contemporary risk scores in predicting outcomes among multi-ethnic or ethnic-specific population.

The GRACE registry initially consisted of 123 hospitals from 14 countries in Europe, North and South America, Australia and New Zealand^20^. This registry initially did not have participation from Asian countries, and consequently the derived original GRACE score was not obtained from Asian patient data^2^. The subsequently updated GRACE 2 registry expanded recruitment to involve 154 hospitals, this time including hospitals from Asia (including China)^20^. Nevertheless, the updated GRACE 2.0 score was only derived from the older registry and was validated in a French cohort^3^. In the Asian context, studies on the GRACE 2.0 score have been performed in ethnically homogenous populations such as in the Japanese^7^, Vietnamese^8^ and Chinese^21^ populations. The Japanese study was a single centre validation study of 412 STEMI patients who had undergone PPCI. This study showed a good AUC of 0.92 in predicting 360-day mortality^7^. The Vietnamese study was performed on 217 patients from a single centre diagnosed with unstable angina, NSTEMI and STEMI. The authors used the score to stratify their patients, but did not specifically study the predictive performance of the GRACE 2.0 score^8^. Fu et al. in China developed the CAMI-NSTEMI score based on 5775 patients from the China Acute Myocardial Infarction (CAMI) registry. They showed that the CAMI-NSTEMI score was superior to that of the GRACE score (AUC 0.81 vs 0.72, p < 0.01) in predicting in-hospital mortality in their Chinese population^21^. We found that the performance of the GRACE 2.0 and modified SMIR scores were similar, be it among all or ethnic-specific AMI patients.

In the modified SMIR score, we found that a higher LVEF was associated with a reduced 1-year all-cause mortality. LVEF is currently not one of the components of the TIMI and GRACE 2.0 scores. LVEF has previously been shown to be associated with an increased mortality in post-MI patients^22^. Therefore, it was worthwhile considering the use LVEF as a variable in risk prediction for AMI patients. Previously, it was difficult to perform a dedicated transthoracic echocardiogram study in the acute setting due to time constraints. However, with the advent of point-of-care echocardiography with portable handheld devices, the LVEF of the patient can be rapidly obtained by the bedside^23^. Future risk scores may consider the use of variables that were previously not readily available.

In addition, notably there are emerging risk stratification tools for AMI patients beyond published risk scores. Emerging approaches, such as metabolomics-based risk stratification, may have a role in future risk stratification beyond current clinically available variables^24,25^. Identified soluble biomarkers, such as those for myocardial fibrosis, may play a role in determining the severity of acute myocardial infarction^26^. Authors have also reported machine-learning based methods for risk stratification of AMI patients using big data approaches, with results that seem to outperform traditional risk models^27,28^. It is not improbable that in the future, risk prediction would incorporate a combination of clinical, haematological, biochemical, echocardiographic and electronic health records-based information, customized to the local context, to provide personalized risk stratification for each AMI patient. Nevertheless, until such technology becomes mature and widely available, and also in areas of practice with resource constraints^29^, traditional risk scores will remain relevant.

### Strengths and limitations

This study used a large national-level database of AMI patients based on mandatory reporting to ensure near-complete case coverage. This also minimized selection bias. Data linkage with the national Death Registry ensured accurate and objective ascertainment of outcomes. Another strength of this study is that this scoring system is based on the contemporaneous treatment population, both in terns of secondary prevention and revascularization.

Nevertheless, we acknowledge several limitations of this study. While Singapore’s ethnically diverse population is ideal for this study, no superiority in using the modified SMIR score compared to the popular and validated GRACE tool was demonstrated. Thus, the scientific and clinical contributions of our findings seem not to be high. Nevertheless, this study fills the literature gap by studying the GRACE 2.0 score in a multi-ethnic Asian population which is currently lacking and demonstrating that GRACE 2.0 is likely to be applicable to other Asian populations that are primarily of Chinese, Malay or Indian origin. As our study focused exclusively on PCI patients alone, our findings cannot be extrapolated to patients without PCI such as the thrombolysis population. However, thrombolysis as a reperfusion strategy is seldom used, at least in Singapore. Although we found that the GRACE 2.0 and modified SMIR scores were able to correctly classify patients broadly into low (< 10%), mid (10–20% and 20–40%) and high (40–60% and > 60%) risk, we were unable to compare the observed mortality for finer subgroups at different predicted risk level due to small sample sizes. Clinicians would need to apply their own clinical judgement should they need more granular risk stratification. Further studies are needed to optimize the performance of the stated scores in predicting 1-year all-cause mortality. Moreover, the points corresponding to categories of some prognostic components, such as age at onset of acute myocardial infarction and the Killip class, are nonlinear, but these components were used for regression models. Therefore, the clinical interpretability of these components needs to be done with cautions.

## Conclusion

In conclusion, we have shown that in a multi-ethnic Asian AMI population with PCI, the modified SMIR score performed similarly to the GRACE 2.0 score.

## Supplementary Information

Supplementary Tables.

## Data Availability

The datasets used in this study are property of the National Registry of Diseases and were collected primarily for internal use. De-identified data can be accessed for public health research purposes after appropriate approval is obtained from the Institutional Review Board and Ministry of Health.
