# Supplementary material for: Comparison of the modified Singapore myocardial infarction registry risk score with GRACE 2.0 in predicting 1-year acute myocardial infarction outcomes
Source: Sci Rep. 2022 Aug 22;12:14270. doi: 10.1038/s41598-022-16523-6 (PMC9395527; doi:10.1038/s41598-022-16523-6)
Supplement: Supplementary file 1 — Supplementary Tables. [file 41598_2022_16523_MOESM1_ESM.docx]

**Supplementary Table 1. Components in the modified SMIR score and predicted risk of 1-year all-cause mortality from the modified SMIR score**

| **Age at onset of AMI** | **Score** |
| --- | --- |
| <40 | 0 |
| 40-49 | 23 |
| 50-59 | 22 |
| 60-69 | 38 |
| 70-79 | 45 |
| 80-89 | 66 |
| >=90 | 100 |
| **History of diabetes** | **Score** |
| No | 0 |
| Yes | 11 |
| **Killip class on admission** | **Score** |
| I | 12 |
| II | 0 |
| III | 16 |
| IV | 53 |
| **Cardiopulmonary resuscitation on admission** | **Score** |
| No | 0 |
| Yes | 56 |
| **Systolic blood pressure on admission** | **Score** |
| <80 | 39 |
| 80-99 | 27 |
| 100-109 | 22 |
| 110-119 | 20 |
| 120-129 | 0 |
| 130-139 | 7 |
| 140-159 | 2 |
| 160-179 | 5 |
| >=180 | 9 |
| **Creatinine on admission** | **Score** |
| 0-34 | 100 |
| 35-69 | 0 |
| 70-105 | 13 |
| 106-140 | 27 |
| 141-176 | 34 |
| 177-353 | 42 |
| >=354 | 43 |
| **Haemoglobin on admission** | **Score** |
| <10 | 40 |
| 10-11 | 36 |
| 12-13 | 14 |
| 14-15 | 1 |
| >=16 | 0 |
| **Left ventricular ejection fraction during hospitalisation** | **Score** |
| <30 | 55 |
| 30-39 | 35 |
| 40-49 | 11 |
| >=50 | 0 |
| **Total score** | **Predicted risk** |
| <=140 | <10% |
| 141-165 | 10-20% |
| 166-180 | 20-30% |
| 181-195 | 30-40% |
| 196-205 | 40-50% |
| 206-220 | 50-60% |
| 221-230 | 60-70% |
| 231-245 | 70-80% |
| 246-270 | 80-90% |
| >270 | 90-100% |

**Supplementary Table 2. Distribution of SMIR patients by the predicted risk of 1-year all-cause mortality from the GRACE 2.0 and modified SMIR scores**

| **Predicted risk based on GRACE 2.0 score** | **Distribution of SMIR patients (%)** |  | **Predicted risk based on modified SMIR score** | **Distribution of SMIR patients (%)** |
| --- | --- | --- | --- | --- |
| <10% | 73.9 |  | <10% | 81.0 |
| 10-20% | 13.4 |  | 10-20% | 7.3 |
| 20-30% | 5.7 |  | 20-30% | 4.4 |
| 30-40% | 2.8 |  | 30-40% | 3.1 |
| 40-50% | 1.7 |  | 40-50% | 1.1 |
| 50-60% | 0.7 |  | 50-60% | 1.3 |
| 60-70% | 0.8 |  | 60-70% | 0.7 |
| 70-80% | 0.2 |  | 70-80% | 0.5 |
| 80-90% | 0.5 |  | 80-90% | 0.5 |
| 90-100% | 0.3 |  | 90-100% | 0.2 |

**Supplementary Table 3.** **Observed 1-year all-cause mortality by predicted risk from the GRACE 2.0 and modfied SMIR scores for each ethnic group**

| **Predicted risk** | **<10%** | **10-40%** | **>40%** |
| --- | --- | --- | --- |
| **Chinese** |  |  |  |
| Observed mortality (%) by the predicted risk from GRACE 2.0 score | 2.8 | 18.0 | 50.0 |
| Observed mortality (%) by the predicted risk from modified SMIR score | 2.9 | 21.0 | 52.8 |
| **Malay** |  |  |  |
| Observed mortality (%) by the predicted risk from GRACE 2.0 score | 3.5 | 31.8 | 66.7 |
| Observed mortality (%) by the predicted risk from modified SMIR score | 3.3 | 32.7 | 81.8 |
| **Indian** |  |  |  |
| Observed mortality (%) by the predicted risk from GRACE 2.0 score | 4.2 | 17.5 | 58.3 |
| Observed mortality (%) by the predicted risk from modified SMIR score | 1.9 | 17.2 | 58.8 |

Patients were further collapsed into broader groups due to small counts.

**Supplementary Table 4. Area under the receiver operator characteristic curve of the GRACE 2.0 and modified SMIR scores for each ethnic group**

|  | **Chinese** | **Malay** | **Indian** |
| --- | --- | --- | --- |
| AUC (95% CI) of GRACE 2.0 score | 0.824  (0.771-0.877) | 0.873  (0.804-0.941) | 0.869  (0.769-0.969) |
| AUC (95% CI) of modified SMIR score | 0.846  (0.800-0.893) | 0.908  (0.862-0.953) | 0.866  (0.773-0.959) |
| p-value of the difference in AUC | 0.196 | 0.248 | 0.914 |
